# Sodium and Health Outcomes: Ascertaining Valid Estimates in Research Studies

**DOI:** 10.1007/s11883-021-00909-4

**Published:** 2021-05-11

**Authors:** Cheryl A. M. Anderson, Erin Delker, Joachim H. Ix

**Affiliations:** 1 University of California San Diego School of Public Health and Human Longevity Science, 9500 Gilman Drive, MC 0628, La Jolla, CA 92093-0628 USA; 2 Department of Medicine, Division of Nephology and Hypertension, University of California San Diego School of Medicine, 9500 Gilman Drive, MC 0628, La Jolla, CA 92093-0628 USA

**Keywords:** Sodium, Dietary reference intakes (DRI), Paradox, Directed acyclic graphs (DAG), Dietary guidelines for Americans (DGA)

## Abstract

**Purpose of Review:**

The dietary reference intake (DRI) for sodium has been highly debated with persuasive and elegant arguments made for both population sodium reduction and for maintenance of the status quo. After the 2015 *Dietary Guidelines Advisory Committee* (DGAC) report was published, controversy ensued, and by Congressional mandate, the sodium DRIs were updated in 2019. The 2019 DRIs defined adequate intake (AI) levels by age–sex groups that are largely consistent with the DRIs for sodium that were published in 2005. Given the overall similarities between the 2005 and 2019 DRIs, one may wonder how the recently published research on sodium and health outcomes was considered in determining the DRIs, particularly, the recent studies from very large observational cohort studies. We aim to address this concern and outline the major threats to ascertaining valid estimates of the relationship between dietary sodium and health outcomes in observational cohort studies. We use tools from modern epidemiology to demonstrate how unexpected and inconsistent findings in these relationships may emerge. We use directed acyclic graphs to illustrate specific examples in which biases may occur.

**Recent Findings:**

We identified the following key threats to internal validity: poorly defined target intervention, poorly measured sodium exposure, unmeasured or residual confounding, reverse causality, and selection bias. Researchers should consider these threats to internal validity while developing research questions and throughout the research process.

**Summary:**

For the DRIs to inform real-world interventions relating to sodium reduction, it is recommended that more specific research questions be asked that can clearly define potential interventions of interest.

## Introduction

Dietary reference intakes (DRIs) have been a cornerstone of United States (US) nutrition policy since 1943 [1]. They impact federally funded nutrition programs and, the recommended population level of sodium can elicit polarizing responses from scientists [2, 3], industry representatives [4, 5], and journalists [6, 7]. Sodium is a nutrient that has been highly debated with persuasive and elegant arguments made for both population sodium reduction [8, 9] and for maintenance of the status quo [10, 11].

After the 2015 *Dietary Guidelines Advisory Committee* (DGAC) report was published [12^••^], controversy ensued, and by Congressional mandate, the sodium DRIs were updated in 2019 [13^••^]. The 2019 adequate intake (AI) levels by age–sex groups are largely consistent with the DRIs published in 2005. The areas of difference are as follows:

- AI of 100 mg/day for infants 0–6 months (decreased from 2005)
- AI of 800 mg/day for children 1–3 years (decreased from 2005)
- AI of 1000 mg/day for children 4–8 years (decreased from 2005)
- AI of 1200 mg/day males and females 9–13 years (decreased from2005)
- AI of 1500 mg/day for adults 51–70 years and adults >70 years (increased from 2005)

In the 2019 DRIs, the tolerable upper level (UL) was no longer used for sodium. This is a point of difference from the DRIs published in 2005 [14] and is based on a revision in methodology such that ULs are driven by toxicological responses [15]. In 2019, instead of ULs for sodium, the chronic disease risk reduction (CDRR) DRIs were set [13^••^]. The CDRR DRIs are a new feature of sodium DRIs. CDRR is the intake level at which reduction in intake is expected to reduce chronic disease risk within an apparently healthy population. The differences between the UL set in the 2005 DRIs and the CDRR set in the 2019 DRIs are as follows:

- a CDRR of 1200 mg/day for children 1–3 years (decrease from 2005 UL)
- a CDRR of 1500 mg/day for children 4–8 years (decrease from 2005 UL)
- a CDRR of 1800 mg/day for males and females 9–13 years (decreased from 2005 UL)

Given the overall similarities between the 2005 and 2019 DRIs, one may wonder how recently published research on sodium and health outcomes was considered in determining the DRIs, particularly the recent studies from very large observational cohort studies [16–21]. And, it may even raise questions as to whether the recommendations from the 2019 committee were simply replicating previous knowledge, or whether it was driven by a lack of certainty in the newly published results.

To answer these questions, it is important to consider that epidemiologic research on sodium and health has sought answers to causal questions such as “will decreased sodium intake reduce risk of cardiovascular disease (CVD)? If so, by how much and for what populations?” Often, these answers can contribute to establishing nutrition guidelines and associated policies, which will subsequently improve population health. When DRIs are being determined, a consensus panel of scientists systematically reviews the literature to evaluate certainty in the presented results and weigh the individual studies based on the potential for bias [22, 23, 24^•^, 25^•^]. Additionally, the DRI committee considers the magnitude and direction of the potential bias and discusses the likelihood that the studies’ conclusions would meaningfully change in the absence of bias. They consider findings from all study designs and must often grapple with the paucity of randomized clinical trials and some inconsistency across the observational epidemiologic studies. Moreover, in epidemiology, there has been a shift towards the use of very large datasets to understand exposure–disease relationships, and the use of larger sample sizes is sometimes misinterpreted as confidence in the results obtained. Although a benefit of using very large datasets is improved *precision* in effect estimation, this does not indicate that these data will yield valid estimates of the relationship between exposure and outcome [26].

Herein, we aim to outline major threats to ascertaining valid estimates of the relationship between dietary sodium and health outcomes in observational cohort studies. We use tools from modern epidemiology to demonstrate how unexpected and inconsistent findings in these relationships may emerge.

### Current Challenges in Estimating Relationships Between Dietary Sodium and Health Outcomes

In observational studies, the main analyses estimate statistical associations between dietary sodium intake and a specific health outcome. Inferring causation from these statistical associations is a difficult task and requires strict assumptions [27]. Nutritional epidemiology, and particularly the study of specific micronutrients, has been criticized as being plagued with methodological issues limiting this inference [28, 29]. In the case of sodium, these doubts contribute to the debates about the recommended intake level for this nutrient and a lack of confidence, by some, in the guidelines [30].

**Fig. 1:**
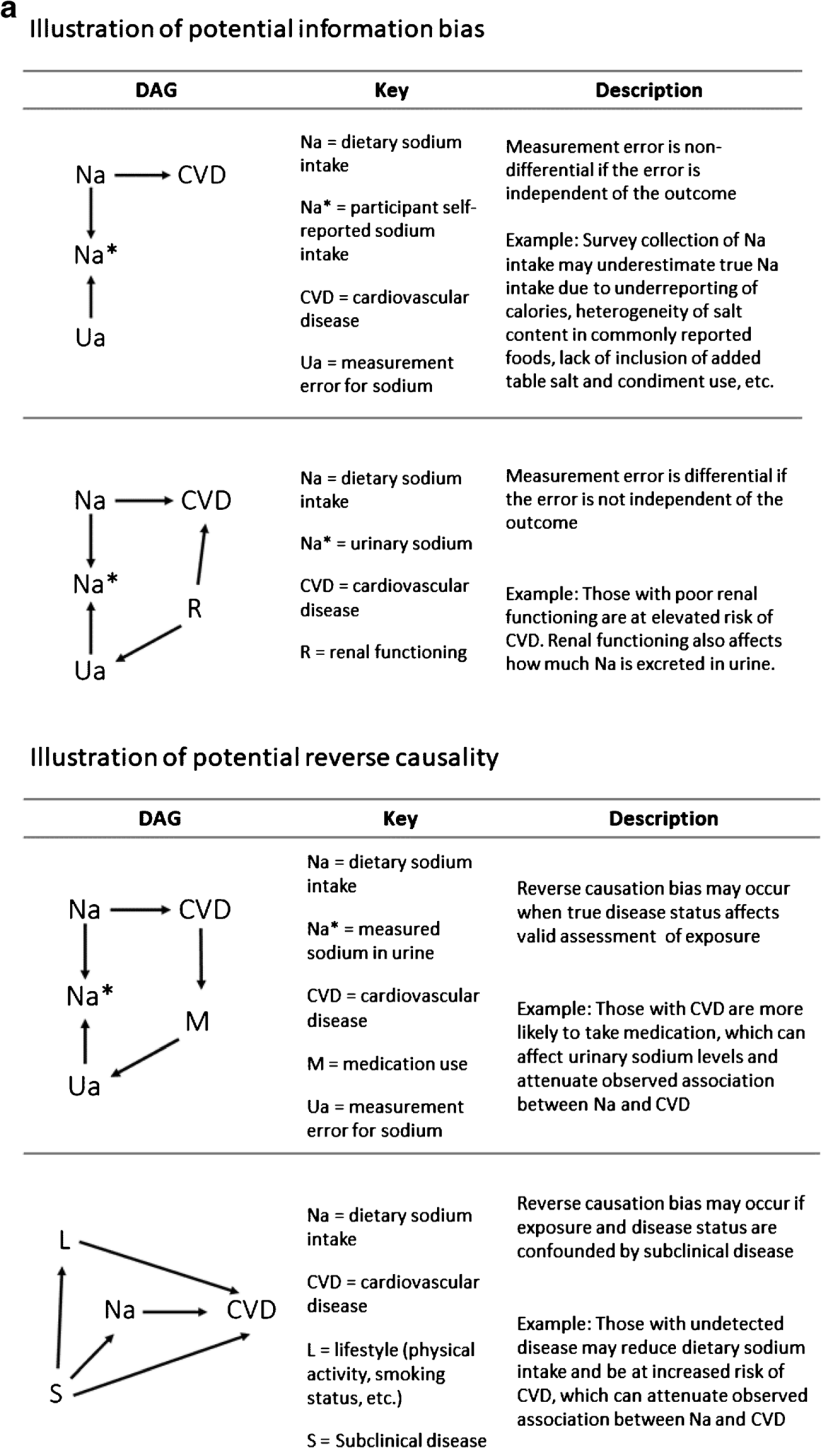

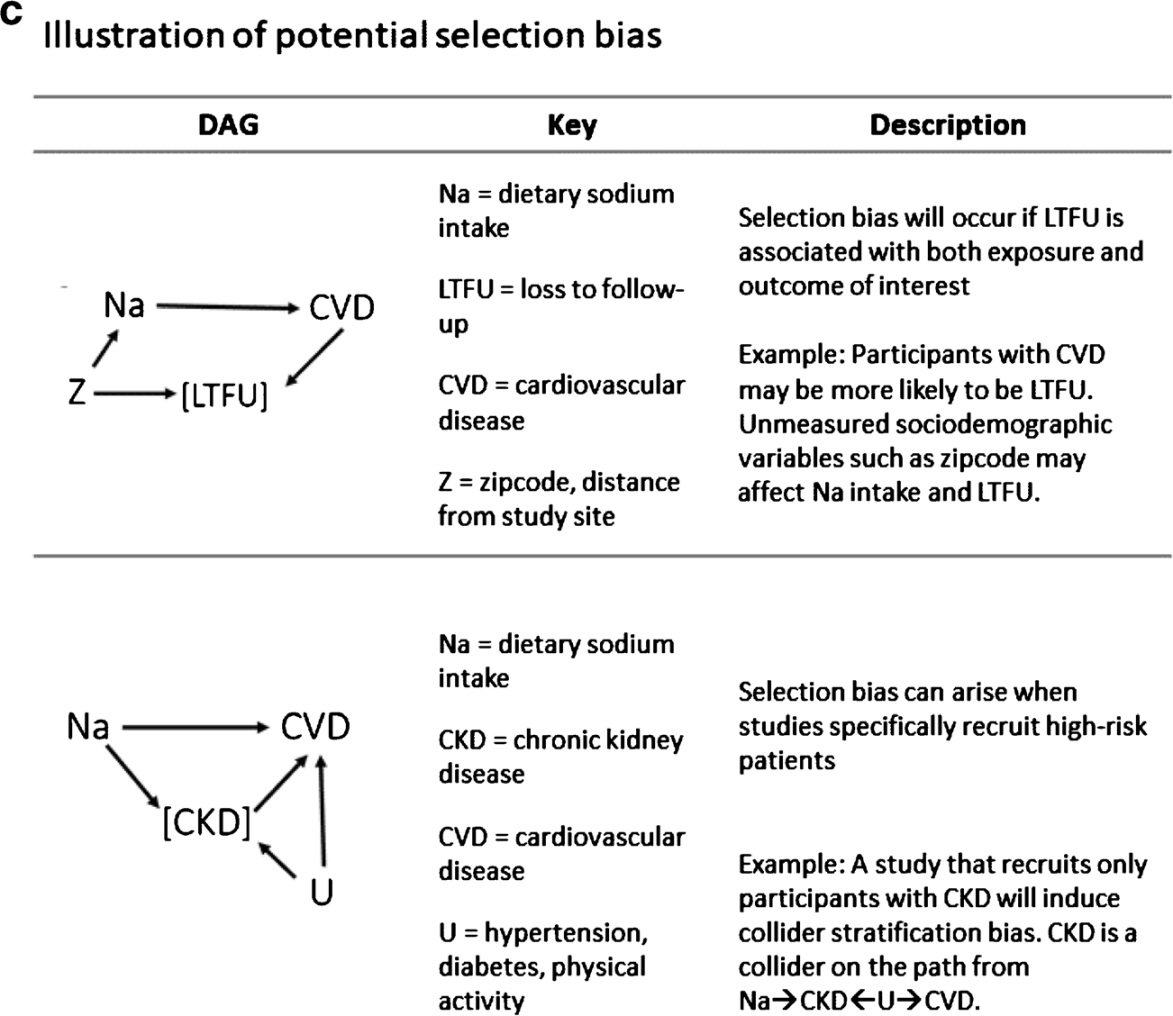
**a** Illustration of potential information bias. **b** Illustration of potential reverse causality. **c** Illustration of potential selection bias

In Table 1, we discuss assumptions in inferring causation from observational data that are the key to the investigation of the effects of sodium intake on health. We illustrate a few of these assumptions (Figs. 1a–c) using causal directed acyclic graphs (DAGs) adapted from figures presented in a textbook by Hernán and Robins (2020) [27]. In brief, a DAG is a graphical representation of the causal effects between variables. They are constructed from a set of edges (arrows) and nodes (variables) based on a priori assumptions about the causal relations among the exposure, outcome, and covariates. An arrow between two variables implies a direct causal effect. Two variables (i.e., *X* and *Y*) may be statistically associated if (1) *X* directly or indirectly causes *Y*, (2) *X* and *Y* share a common cause (i.e., confounding variable), or (3) a descendent of *X* and *Y* (i.e., collider) has been conditioned on [27, 31–33].

**Table 1:** Key assumptions in inferring causation from observational data in the context of sodium intake and CVD

**1. Exposure is well-defined**	
A key assumption for estimating causal effects from observational data is that the exposure is well defined and that the observed and counterfactual outcomes are clear [34]. More so, we assume that the effect of an exposure, such as dietary sodium intake, on a specific outcome will be the same regardless of how the exposure is modified [34]. For example, an individual may need to reduce their total dietary sodium intake by 30% to meet DRI levels. There are several ways to reduce total dietary sodium intake (replacement of high-sodium foods, caloric reduction, elimination of table salt, etc.). It is straightforward to challenge the assumption that each of these approaches (i.e., hypothetical interventions) will have consistent causal effects on the outcome of interest. Dietary sodium intake is a complex exposure because it is, almost always, consumed among other nutrients.	
**2. Exposure is measured without error**	
Issues relating to measurement of dietary sodium intake are widely acknowledged [28, 29, 35]. In brief, there are two primary modes of collection: measurements in urine specimens and dietary surveys. Dietary surveys include food frequency questionnaires (FFQs), food diaries, and 24-h recall. These assessments are inexpensive and with repeated measurements could capture an individual’s usual sodium intake; however, they are vulnerable to recall and reporting biases. Additionally, processing data from these surveys requires researchers to estimate sodium levels from food composition. Accuracy in these conversions is limited due to heterogeneity in sodium content across commonly reported foods. Moreover, sodium intake is likely underreported when condiments, table salt, and sodium used when cooking is not included in survey items (Fig. 1a, top). An advantage of urinary collection methods is that they are objective measurements of sodium recovered in urine. Though, to reduce participant burden and study costs, large studies sometimes rely on a single collection of urine via an overnight or spot urine collection, which may vary by time of day and time since consumption of sodium. Bias could be introduced if estimating equations are used to deduce 24 h excretion from a spot urine collection. In addition, due to day-to-day variation in sodium intake, one measurement may not accurately represent usual intake. This random error affects the measurements of all participants under study (Fig. 1a, top). An additional concern is a circumstance in which measurement error in sodium is differential by study outcome. This will occur if the amount of sodium excreted in urine varies by variables associated with CVD risk, such as medication use or kidney function when no longer in steady state. As depicted in the bottom half of Fig. 1a, a third variable, kidney function, is creating a “back door path” (i.e., biasing pathway) due to its association with sodium measurement error and CVD (Fig. 1a, bottom).	
**3. Confounding variables are measured and adjusted for**	
Another key assumption for causal inference from observational studies is that there are no unmeasured variables that affect risk of the outcome and are also differentially distributed by exposure status. For example, when we compare disease risk among participants that have the greatest reported dietary sodium intake vs. those with the least, we assume that this is a valid comparison or that these groups are exchangeable (i.e., conditional exchangeability). This assumption of conditional exchangeability is challenged when there are large differences in unmeasured (or measured) confounding variables across exposure categories. Major differences in the distribution of sodium intake by key demographic variables may be difficult to fully address with traditional methods of adjustment, resulting in residual confounding. Dietary sodium intake is correlated with overall dietary pattern and total caloric intake. Thus, it requires careful work to isolate the specific effect of sodium on a disease outcome.	
**4. No reverse causality**	
Absence of reverse causality implies that the exposure affects the outcome and not vice versa. For example, this is often assumed in a prospective cohort study because the exposure is measured at baseline when disease is absent, and participants are followed for incident disease. However, in Fig. 1b, we illustrate two scenarios in which reverse causation bias may occur. In the first example, participants with pre-existing risk factors or subclinical disease at baseline may use medications that affect valid assessment of the exposure (Fig. 1b, top). In the second example, individuals with subclinical disease are at increased risk to develop the study outcome and may have already reduced their sodium intake via change in diet. As such, a study of these participants would underestimate sodium intake in the disease group and consequently underestimate the true effect of sodium on a health outcome (Fig. 1b, bottom).	
**5. Absence of selection bias**	
The way in which individuals are selected into study analyses can contribute to biased and unexpected results. Studies of sodium and CVD that preferentially recruit sicker patients (i.e., T2D and CKD) either by design or because of recruitment procedures can show the counterintuitive result that increased sodium is protective for CVD. For example, increased sodium intake is associated with greater risk of chronic kidney disease (CKD) which is, in turn, associated with increased risk of CVD. Additionally, CKD and CVD share many risk factors. Thus, conducting an analysis within a stratum of participants with CKD is, by design, conditioning on a common cause of exposure and outcome (i.e., collider stratification bias). Within this stratum, the study data will underestimate sodium intake and overestimate CVD incidence compared to the target population. This could result in unexpected null associations, or inverse associations between sodium and CVD (Fig. 1c bottom). Missing data due to loss to follow-up (LTFU) may also result in selection bias if the LTFU is associated with variables under study. For example, if the sickest participants are those that are most likely to be LTFU, then the analytic sample would be deplete of those with the highest exposure and most disease, resulting in a bias towards the null (Fig. 1c, top).	
**6. Findings are generalizable to target population**	
Lastly, effect estimates may differ across sub-populations. Several studies regarding sodium consumption and health include only high-risk patients (i.e., end-stage renal disease and type 2 diabetes mellitus). Although it is necessary to learn about the health effects of sodium in these clinical populations, the findings may not be generalizable to the US population. Thus, guidelines directed towards the US population usually do not prioritize these findings.

Increasingly, DAGs are being used to help depict different causal structures, thus forcing researchers to be explicit about the research question and the underlying assumptions about how the variables of interest are related. This approach facilitates communication within the research community giving us a framework around which we can align. Additionally, DAGs facilitate appropriate selection of covariates for regression analyses and help elucidate potential sources of bias.

## Discussion and Conclusions

We aimed to outline major threats to ascertaining valid estimates of the relationship between dietary sodium and health outcomes in observational cohort studies. We use directed acyclic graphs to illustrate specific examples in which biases may occur. These are tools that can be used throughout the research process to inform which variables should be measured in research studies, what variables should be adjusted for in our multivariable analyses, and how the procedures used to select participants into studies affect internal validity of study results. They can also be used alongside bias quantification methods [22, 23, 24^•^, 25^•^] to estimate the magnitude and the direction of the bias present.

The key threats to internal validity we have identified in this paper are as follows:

- poorly defined target intervention
- poorly measured sodium exposure
- unmeasured or residual confounding
- reverse causality
- selection bias

Researchers should consider these threats to internal validity while developing research questions and throughout the research process. A well-defined question with a clearly articulated target intervention can be more easily translated to nutritional policy. Other threats to validity can be eased during the study design process. For example, using multiple modes of sodium measurement such that findings can be contrasted within the same study sample will inform the extent to which measurement error is biasing results. Bias due to confounding and reverse causality can be eased by measurement of auxiliary variables. Developing a DAG in collaboration with subject area experts can be used to identify which variables need to be measured and then subsequently adjusted for in statistical analyses.

We also highlight the importance of clearly defining a target population for which the study results should generalize to. Threats to external validity, too, have implications for nutritional policy makers. Studies of sodium and disease in clinical and high-risk populations are beneficial in understanding physiologic mechanisms at play as well as targeted interventions for these groups. These studies should not be prioritized, however, in informing national dietary guidelines—which are focused on establishing recommendations for health promotion and disease prevention across the US. It is imperative that observational research informing national guidelines includes representation of all population subgroups and that the study population is representative of the general US population.

## Conclusions

Despite strong opinions about the usefulness of nutritional epidemiology [29, 36, 37], and the labeling of this field as flawed [29], it may be more productive and informative to think through how the limitations of the methods employed in these studies affect their conclusions. This can guide us to understand the implications of published analyses, regardless of the size of the dataset, and help inform well designed studies that can be used to set sodium policies.

## References

[1] National Research Council (US) Subcommittee on the Tenth Edition of the Recommended Dietary Allowances. Recommended dietary allowances 10^th^ Edition. National Academies Press, Washington DC. 1989. 25144070

[2] Johnston BC, Zeraatkar D, Han M, Vernooij RWM, Valli C, El Dib R, Marshall C, Stover P, Fairweather-Taitt S, Wojcki G (2019). Unprocessed red meat and processed meat consumption: dietary guideline recommendations from the nutritional recommendations (NutriRECS) consortium. Ann Intern Med.

[3] Is eating red meat OK afterall? Probably not. https://news.harvard.edu/gazette/story/2019/11/clearing-up-the-confusion-over-red-meat-recommendations/ Interview with Dr. Frank Hu by Alvin Powell. Last accessed on September 7, 2020.

[4] Nutrition Coalition. Guidelines fall short of best scientific practices. https://www.nutritioncoalition.us/2020-dietary-guidelines-info/dietary-guidelines-fail-to-meet-review-standards. .

[5] Nutrition Coalition and it’s nonprofit affiliate National Alliance for Better Nutrition (NABN). For a healthier America we need dietary guidelines based on sound scientific evidence.https://forbetterdietaryguidelines.org/ Last accessed on September 7, 2020.

[6] The Big Fat Surprise (2014). Why butter, meat and cheese belong in a healthy diet, by Nina Teicholz.

[7] Food Politics: Dietary reference intakes are now political??? by Marion Nestle, https://www.foodpolitics.com/2018/01/dietary-reference-intakes-are-now-political-2/ Last accessed on September 7, 2020.

[8] Appel LJ, Angel SY, Cobb LK, Limper H, Nelson DE, Samet JM, Brownson RC (2012). Population-wide sodium reduction: the bumpy road from evidence to policy. Ann Epidemiol.

[9] Kaplan NM (2000). The dietary guideline for sodium: should we shake it up?. No Am J Clin Nutr.

[10] McCarron D (2000). The dietary guideline for sodium: should we shake it up?. Yes Am J Clin Nutr.

[11] McCarron D, Drüeke T, Stricker E (2010). Science trumps politics: urinary sodium data challenge US dietary sodium guideline. Am J Clin Nutr.

[12] •• Dietary Guidelines Advisory Committee. 2015. Scientific report of the 2015 Dietary Guidelines Advisory Committee: advisory report to the Secretary of Agriculture and the Secretary of Health and Human Services. U.S. Department of Agriculture, Agricultural Research Service, Washington, DC. https://health.gov/sites/default/files/2019-09/Scientific-Report-of-the-2015-Dietary-Guidelines-Advisory-Committee.pdf. Last accessed September 8, 2020. **This advisory report helps to inform the federal government of the body of scientific evidence on topics related to diet, nutrition, and health. The advisory report is not the Dietary Guidelines policy or a draft of the policy. The 2015–2020 Dietary Guidelines were designed to help Americans eat a healthier diet. It is intended for policymakers and health professionals and outlines how people can improve their overall eating patterns.**

[13] •• National Academies of Sciences, Engineering, and Medicine. 2019. Dietary reference intakes for sodium and potassium. Washington, DC: The National Academies Press. 10.17226/25353. **Dietary reference intakes (DRIs) are the foundation for United States nutrition policy and are adhered to by all federally funded nutrition programs. In 2019, the DRIs for sodium and potassium were updated.** 30844154

[14] Institute of Medicine (2005). Dietary reference intakes for water, potassium, sodium, chloride, and sulfate.

[15] National Academies of Sciences, Engineering, and Medicine. 2017. Guiding principles for developing dietary reference intakes based on chronic disease. Washington, DC: The National Academies Press. 10.17226/24828. 29200241

[16] Mente A. O’Donnell M, Rangarajan, McQueen M, Dagenais G, Wielgosz A. Urinary sodium excretion, blood pressure, cardiovascular disease, and mortality: a community-level prospective epidemiological cohort study 2018; 392(10146): 496–506. 10.1016/S0140-6736(18)31376-X 30129465

[17] Mente A, O'Donnell M, Rangarajan S, et al. Associations of urinary sodium excretion with cardiovascular events in individuals with and without hypertension: a pooled analysis of data from four studies. *Lancet (London, England)*. 2016; 388: 465–475. 10.1016/S0140-6736(16)30467-6 27216139

[18] Mente A, O'Donnell MJ, Rangarajan S, McQueen MJ, Poirier P, Wielgosz A, Morrison H, Li W, Wang X, di C, Mony P, Devanath A, Rosengren A, Oguz A, Zatonska K, Yusufali AH, Lopez-Jaramillo P, Avezum A, Ismail N, Lanas F, Puoane T, Diaz R, Kelishadi R, Iqbal R, Yusuf R, Chifamba J, Khatib R, Teo K, Yusuf S (2014). Association of urinary sodium and potassium excretion with blood pressure. N Engl J Med.

[19] O’Donnell M, Mente A, Rangarajan S, McQueen MJ, Wang X, Liu L, Yan H, Lee SF, Mony P, Devanath A (2014). For the PURE investigators. Urinary sodium and potassium excretion, mortality, and cardiovascular events. N Engl J Med.

[20] Oparil S (2014). Low sodium intake – cardiovascular health benefit or risk?. New Engl J Med.

[21] Tan M, He F, MacGregor GA (2018). Salt and cardiovascular disease in PURE: a large sample size cannot make up for erroneous estimations. J Renin-Angiotensin-Aldosterone Syst.

[22] Sterne JAC, Savović J, Page MJ, Elbers RG, Blencowe NS, Boutron I, Cates CJ, Cheng H-Y, Corbett MS, Eldridge SM, Hernán MA, Hopewell S, Hróbjartsson A, Junqueira DR, Jüni P, Kirkham JJ, Lasserson T, Li T, McAleenan A, Reeves BC, Shepperd S, Shrier I, Stewart LA, Tilling K, White IR, Whiting PF, Higgins JPT (2019). RoB 2: a revised tool for assessing risk of bias in randomised trials. BMJ.

[23] Higgins JPT, Sterne JAC, Savović J, Page MJ, Hróbjartsson A, Boutron I, Reeves B, Eldridge S. A revised tool for assessing risk of bias in randomized trials In: Chandler J, McKenzie J, Boutron I, Welch V (editors). Cochrane methods. *Cochrane Database of Systematic Reviews* 2016, Issue 10 (Suppl 1).10.1002/14651858.CD201601.

[24] • National Institutes of Health National Heart Lung and Blood Institute. Study quality assessment tools. https://www.nhlbi.nih.gov/health-topics/study-quality-assessment-tools. Last accessed September 8, 2020. **When DRIs are being determined, a consensus panel of scientists systematically reviews the literature to evaluate certainty in the presented results and weigh the individual studies based on the potential for bias. This is an example of a tool that is commonly used for observational studies.**

[25] • Page MJ, McKenzie J, Higgins JPT. Tools for assessing risk of reporting biases in studies and syntheses of studies: a systematic review. BMJ Open. 2020; 8(3): 10.1136/bmjopen-2017-019703. **Findings from this study are that there are several limitations of existing tools for assessing risk of reporting biases, in terms of their scope and guidance for reaching risk of bias judgements and measurement properties.** 10.1136/bmjopen-2017-019703 PMC5857645 29540417

[26] Mooney SJ, Westreich DJ, El-Sayed AM (2015). Epidemiology in the era of big data. Epidemiology..

[27] Hernán M, Robins J (2019). Causal inference.

[28] Satija A, Yu E, Willett WC, Hu FB (2015). Understanding nutritional epidemiology and its role in policy. Adv Nutr.

[29] Ioannidas J (2018). The challenge of reforming nutrition epidemiologic research. JAMA..

[30] Prentice RL, Huang Y (2018). Nutritional epidemiology methods and related statistical challenges and opportunities. Stat Theory Relat Fields.

[31] Glymour MM. Using causal diagrams to understand common problems in social epidemiology. In J. M. Oakes & J. S. Kaufman (Eds.), Methods in social epidemiology (p. 393–428). Jossey-Bass/Wiley.

[32] Shrier I, Platt RW (2008). Reducing bias through directed acyclic graphs. BMC Med Res Methodol.

[33] Sauer B, VanderWeele TJ. Use of directed acyclic graphs. Agency for Healthcare Research and Quality (US); 2013. https://www.ncbi.nlm.nih.gov/books/NBK126189/. .

[34] Hernán MA (2016). Does water kill? A call for less casual causal inferences. Ann Epidemiol.

[35] Cobb LK, Anderson CAM, Elliott P, Hu FB, Liu K, Neaton JD, Whelton PK, Woodward M, Appel LJ, American Heart Association Council on Lifestyle and Metabolic Health (2014). Methodological issues in cohort studies that relate sodium intake to cardiovascular disease outcomes: a science advisory from the American Heart Association. Circulation..

[36] Trepanowski JF, Ioannidas J (2018). Perspective: limiting dependence on nonrandomized studies and improving randomized trials in human nutrition research: why and how. Adv Nutr.

[37] Hu FB, Willett W (2018). Current and future landscape of nutritional epidemiologic research. JAMA.

